# A Mysterious Case of Encephalitis: Diagnostic Challenge and Our Clinical Reasoning

**DOI:** 10.7759/cureus.29051

**Published:** 2022-09-11

**Authors:** Bhavani Nagendra Papudesi, Maija Adourian, Srikrishna V Malayala, Sai Deepika Potluri, Haroon Chaudhry, Mathew Mathew

**Affiliations:** 1 Internal Medicine, Suburban Community Hospital, Philadelphia, USA; 2 Medicine, Philadelphia College of Osteopathic Medicine, Philadelphia, USA; 3 Internal Medicine, Temple University Hospital, Philadelphia, USA; 4 Internal Medicine, Reading Hospital - Tower Health, Reading, USA

**Keywords:** anti-nmda receptor encephalitis, methylprednisolone, nmda, cerebrospinal fluid (csf), altered mental status in young, critical reasoning, dilemma, rare cause of altered mental status, anti-nmdar encephalitis

## Abstract

Acute psychotic symptoms in young patients are frequently attributed to toxic or infectious causes. After ruling out the most common causes, obtaining a firm diagnosis becomes challenging. In this case report, we present the case of a young woman who presented with acute psychosis after returning from a five-day vacation in Mexico. We treated this as a case of cerebral spinal fluid (CSF)-negative anti-N-methyl-D-aspartate receptor (NMDAR) encephalitis, as testing for CSF-NMDA receptor IgG antibodies was negative, and the absence of anti-NMDAR IgG antibodies does not rule out this autoimmune encephalitis. Moreover, IV methylprednisolone remarkably improved our patient’s mental status and behavior. Anti-NMDAR encephalitis manifests itself in a variety of ways. As a result, providers must maintain a high level of suspicion based on their clinical assessment, as delays in labs or failure to diagnose early based on the clinical presentation can lead to delays in treatment with which this severe immune-mediated paraneoplastic condition can quickly escalate and have worse consequences. We describe our thought process behind our clinical judgment toward this atypical scenario to contribute to identifying this condition early on in the complex clinical presentation.

## Introduction

Immune-mediated conditions are always diagnosed after ruling out common causes like infectious, neoplastic, traumatic, and toxic etiologies. Abstracting a firm diagnosis during the early stages of autoimmune conditions is always time-consuming. It becomes challenging when physical exam findings do not concord with the imaging findings or lab values. The suffering and damage to the patient will depend on the delay in identifying the etiology and providing the correct treatment. Hence, a physician must connect the dots of clinical presentation, determine the exact etiology, and treat it appropriately in the setting of negative imaging or lab findings. This comes with a high index of suspicion, extensive medical knowledge, extraordinary clinical examination skills, and experience. We report a case of a young woman who presented with acute psychosis after returning from a five-day vacation in Mexico. We treated her empirically as a case of cerebral spinal fluid (CSF)-negative anti-N-methyl-D-aspartate receptor (NMDAR) encephalitis based on clinical suspicion and correlating with incidental imaging findings of an ovarian lesion as mentioned in literature [1]. Our clinical judgment was helpful for the patient and resolved her symptoms without causing any harm to her. Here, we explain our clinical reasoning and judgment toward establishing the diagnosis and empiric treatment for this acute and atypical presentation.

This case was previously presented as a poster at the 2021 Pennsylvania-ACP Southeastern Chapter Virtual Poster Day and Doctor's Dilemma competition on October 23, 2021.

## Case presentation

 A 23-year-old African American female without significant past medical history or known psychiatric conditions was admitted to the hospital's emergency department (ED) with new-onset acute psychosis. Two days prior, she returned from a trip to Cancún, Mexico. Family history and surgical history were non-contributory. She was not on any home medications. She lived with her family, was sexually active, and used marijuana weekly. In the ED, she was afebrile, hemodynamically stable, oriented only to herself, trying to get out of bed and speaking inappropriate words, and showed no signs of meningitis on physical examination. The complete blood count (CBC) was significant for elevated white blood cells (WBCs) at 20.6x103/L and elevated absolute neutrophil count (ANC) at 16.6x103/L. Urinalysis was positive for ketones, and a urine toxicology screen was positive only for cannabinoids.

**Figure 1:**
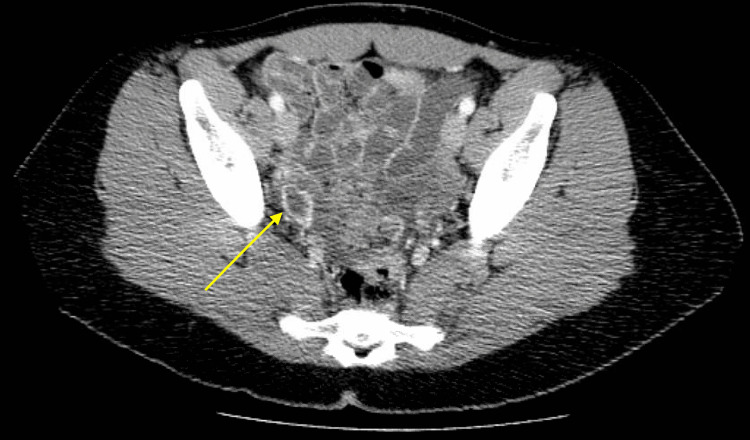
Computed tomography scan of the abdomen and pelvis, which revealed a crenulated right ovarian lesion (indicated by the yellow arrow)

Negative laboratory workup included infectious causes like human immunodeficiency virus (HIV), herpes simplex virus (HSV-1, HSV-2), rapid plasma reagin (RPR) for syphilis, Neisseria (B.) gonorrhea, Chlamydia (C.) trachomatis, Lyme's disease, West Nile virus, and coronavirus disease 2019 (COVID-19). Negative labwork for toxic causes included ethanol, tricyclic antidepressants, oxycodone, propoxyphene, and salicylates. Negative labwork for metabolic causes included a complete metabolic panel, ammonia, lipid panel, and ferritin. Negative labwork for immune-mediated etiologies included anti-nuclear antibodies (ANA), cerebrospinal fluid anti-N-methyl-D-aspartate receptor (NMDAR) immunoglobulin G (IgG) antibodies, and CSF oligoclonal bands. Negative labwork for neoplastic causes included alpha-fetoprotein (AFP). The results of computed tomography (CT) scan of the head without contrast, magnetic resonance imaging (MRI) of the brain with IV contrast, and electroencephalography (EEG) were also negative. However, a CT scan of the abdomen and pelvis revealed a crenulated right ovarian cyst measuring 2.5 cm x 1.6 cm, with a yellow-colored arrow in Figure 1. Ovarian teratomas are commonly associated with anti-NMDAR encephalitis, a paraneoplastic syndrome that presents with a change in mental status. By having a high level of clinical suspicion for this potentially fatal condition, we initiated prompt treatment with IV methylprednisolone 1 gram, once daily for five days. Neurology was consulted, who recommended ordering a full encephalitis panel, thyroid profile, and thyroid antibodies. We have given one dose of IV methylprednisone before the neurologist's evaluation. They agreed with our treatment plan and eventually agreed that there had been an improvement in the patient's delirium with steroids. Our patient demonstrated marked improvement with this treatment and ultimately was discharged home without needing to transfer to an inpatient psychiatric facility.

## Discussion

Young patients' acute changes in mental status are often attributed to toxic or infectious etiologies. After a regular workup of our patient's acute psychotic episode, some differential diagnoses were toxic encephalopathy, infective encephalitis, brief psychotic disorder, systemic lupus erythematosus (SLE) psychosis, and paraneoplastic encephalitis. Toxic encephalopathy could explain memory loss and personality changes. However, it would not explain the delay in symptom onset or the extended symptom duration almost seven days after the onset. The elevated WBC and ANC support infectious encephalitis, but laboratory workup for HIV, HSV-1, HSV-2, RPR, N. gonorrhea, C. trachomatis, Lyme's, West Nile, severe acute respiratory syndrome coronavirus 2 (SARS-Cov-2), and CSF analysis was negative. A brief psychotic disorder could explain the hallucinations and delusions. Still, psychiatric consultation did not diagnose this due to their high suspicion of an underlying medical condition, and antipsychotics did not attenuate her symptoms. SLE-induced psychosis warranted workup given the high prevalence of SLE in African American women of childbearing age. However, ANA, which has 98% sensitivity for SLE, was negative.

After discovering her ovarian lesion, she was worked up for anti-NMDA receptor encephalitis, but the results took almost more than a week. We started empiric treatment with steroids to avoid delays in treatment while waiting for lab results. Though empiric treatment with IV methylprednisolone caused marked clinical improvement, serum and CSF anti-NMDAR antibodies returned with a negative test result. After a thorough workup ruled out all other possible causes, we diagnosed this case as CSF-negative anti-NMDAR encephalitis.

Anti-NMDAR encephalitis is an autoimmune disorder with a wide range of symptoms that was first described in 2005. Both neurological and psychiatric clinical features are seen, and some of the most common ones are mentioned in Table 1. Some studies analyzed the effects of antibodies on neuronal cultures as determined by quantitative analysis of NMDA-receptor clusters. They state that the pathogenesis of these clinical features is mediated by antibodies [1].

**Table 1:** Symptoms reported in anti-NMDA receptor encephalitis NMDA: N-methyl-D-aspartate

Psychiatric	Neurological
Hallucinations	Seizure
Delusions	Abnormal behaviors
Agitation	Hypoventilation
Confusion	Abnormal postures
Labile mood, mania	Memory deficits
Insomnia	Mutism
Depression	Coma
Eating disorders	Rigidity/catatonia
Panic attacks	Gait disturbance
Cognitive decline	Lethargy
Sexual disinhibition	Parkinsonism
Aggression	Speech abnormalities
Suicidal ideation	

However, the absence of the anti-NMDAR IgG antibody does not rule out a diagnosis of other forms of autoimmune encephalitis. In young women, case reports indicate an association between the incidence of anti-NMDAR encephalitis and paraneoplastic syndrome in ovarian teratomas [1,2]. Still, a few reports aimed at identifying the prevalence of anti-NMDAR encephalitis indicated that the rate of NMDAR encephalitis in ovarian teratomas is low, and NMDAR encephalitis patients have smaller teratomas than non-NMDAR encephalitis patients. They have concluded that further studies are needed to understand the timing of anti-NMDA receptor antibodies in teratomas and the development of NMDAR encephalitis [3]. The sensitivity of anti-NMDA receptor antibody testing is higher in CSF than in serum. Higher antibody titers in CSF and serum were associated with underlying teratoma as opposed to no tumor. Patients with high titers were also associated with poorer outcomes. The titer change in CSF was more closely related to relapses than in serum [4]. Early surgical tumor removal and immunotherapy treatment have resulted in a favorable prognosis in patients with ovarian teratomas [5]. Even though many patients do not quickly respond to first-line immunotherapy, our patient showed significant improvement in mentation and behavior after receiving IV methylprednisolone 1 gram once daily for five days.

A few case reports and literature reviews suggest that the Japanese Encephalitis (JE) virus may be a clinically significant cause of anti-NMDAR encephalitis in children. Patients with JE-induced anti-NMDAR encephalitis present with symptoms similar to those with primary anti-NMDAR encephalitis. In these patients, the diagnosis of JE was made based on positive JE antibody test results in serum, cerebrospinal fluid, and antibodies against NMDAR detected in serum and CSF [6]. Most patients with JE-induced anti-NMDAR encephalitis showed an excellent response to first-line immunotherapies. Some studies have reported that infectious pathogens co-exist with antineuronal antibodies in patients with autoimmune encephalitis [7]. The herpes simplex virus has been reported to be associated with anti-NMDA receptor encephalitis [8], and they may also benefit from immunotherapy.

In some cases, anti-NMDA receptor IgG antibodies against the GluN1 subunit were detected in serum but not in CSF. In this situation, anti-NMDA receptor encephalitis diagnosis was made with (18F)fluorodeoxyglucose positron emission tomography (FDG-PET), which showed pronounced relative hypermetabolism of association cortices and a relative hypometabolism of the primary cortices. Treatment with steroids, in this case, also lead to prompt recovery [9].

## Conclusions

The presentation of anti-NMDAR encephalitis is highly variable. The diagnostic parameters are also not very definitive, especially when it comes to ruling it out with negative lab tests. The horizon of differential diagnoses is vast for treating an acute psychotic episode in a young patient with no past medical or psychiatric history. Therefore, providers must maintain high suspicion and adopt a critical and comprehensive thought process for diagnosing the etiology. In general, physicians should be able to link the clinical presentation with the most common possible etiologies and treat them appropriately in the setting of negative imaging or situations where lab results will take a long time. This requires a high index of suspicion, extensive medical knowledge, extraordinary clinical examination skills, and experience. Clinical judgment and decision-making are critical and should precede diagnostic studies in atypical cases such as this. If misdiagnosed, these patients could get mistreated by assuming a psychiatric diagnosis. If an accurate diagnosis or treatment is delayed, this severe immune-mediated paraneoplastic condition can quickly escalate and have life-threatening consequences.

## References

[1] Dalmau J, Gleichman AJ, Hughes EG (2008). Anti-NMDA-receptor encephalitis: case series and analysis of the effects of antibodies. Lancet Neurol.

[2] Chiu HC, Su YC, Huang SC, Chiang HL, Huang PS (2019). Anti-NMDAR encephalitis with ovarian teratomas: review of the literature and two case reports. Taiwan J Obstet Gynecol.

[3] Li JH, Milla SS, Gombolay GY (2022). Rate of anti-NMDA receptor encephalitis in ovarian teratomas. Neuropediatrics.

[4] Gresa-Arribas N, Titulaer MJ, Torrents A (2014). Antibody titres at diagnosis and during follow-up of anti-NMDA receptor encephalitis: a retrospective study. Lancet Neurol.

[5] Gomes Ferreira M, Lapresa Alcalde V, García Sánchez MH, Hernández Hernández L, Doyague Sánchez MJ (2018). Successful treatment of anti-NMDA receptor encephalitis with early teratoma removal and plasmapheresis: a case report. Medicine (Baltimore).

[6] Pastel H, Chakrabarty B, Saini L, Kumar A, Gulati S (2017). A case of anti- N-methyl-D-aspartate (NMDA) receptor encephalitis possibly triggered by an episode of Japanese B encephalitis. Neurol India.

[7] Lin JJ, Lin KL, Chiu CH, Hsia SH, Wang HS, Chou IJ, Lin YT (2014). Antineuronal antibodies and infectious pathogens in severe acute pediatric encephalitis. J Child Neurol.

[8] Prüss H, Finke C, Höltje M (2012). N-methyl-D-aspartate receptor antibodies in herpes simplex encephalitis. Ann Neurol.

[9] Endres D, Rauer S, Kern W (2019). Psychiatric presentation of anti-NMDA receptor encephalitis. Front Neurol.

